# Supplementary material for: Modifiable and nonmodifiable factors associated with anxiety, depression, and stress after one year of the COVID-19 pandemic
Source: PLoS One. 2023 Mar 23;18(3):e0283422. doi: 10.1371/journal.pone.0283422 (PMC10035880; doi:10.1371/journal.pone.0283422)
Supplement: S1 File — (DOCX) [file pone.0283422.s001.docx]

# Supplementary Material 1: Univariate regressions

## Factors associated with anxiety scores

S1 Table. Univariable regression coefficients for non-modifiable factors associated with anxiety scores (square root transformed) among Bangladeshi adults

| **Explanatory variables** | **Coefficient (Standard Error)** | **Coefficient (Standard Error)** | **Coefficient (Standard Error)** | **Coefficient (Standard Error)** | **Coefficient (Standard Error)** | **Coefficient (Standard Error)** | **Coefficient (Standard Error)** | **Coefficient (Standard Error)** | **Coefficient (Standard Error)** |
| --- | --- | --- | --- | --- | --- | --- | --- | --- | --- |
| **Sex** |  |  |  |  |  |  |  |  |  |
| ***Male (ref)*** |  |  |  |  |  |  |  |  |  |
| ***Female*** | .24 **(.08) |  |  |  |  |  |  |  |  |
| **Age (in years)** |  | -.03*** (.004) |  |  |  |  |  |  |  |
| **Education** |  |  |  |  |  |  |  |  |  |
| ***Postgraduate (ref)*** |  |  |  |  |  |  |  |  |  |
| ***Nonformal*** |  |  | .004 (.209) |  |  |  |  |  |  |
| ***SSC/Equivalent*** |  |  | .138 (.171) |  |  |  |  |  |  |
| ***HSC/Equivalent*** |  |  | .551*** (.103) |  |  |  |  |  |  |
| ***Bachelor/Equivalent*** |  |  | .411*** (.105) |  |  |  |  |  |  |
| **History of mental illness** |  |  |  |  |  |  |  |  |  |
| ***No (ref)*** |  |  |  |  |  |  |  |  |  |
| ***Yes*** |  |  |  | .895*** (.126) |  |  |  |  |  |
| ***Prefer Not to Say*** |  |  |  | .671** (.222) |  |  |  |  |  |
| **Division** |  |  |  |  |  |  |  |  |  |
| ***Dhaka (ref)*** |  |  |  |  |  |  |  |  |  |
| ***Barisal*** |  |  |  |  | .222 (.159) |  |  |  |  |
| ***Rajshahi*** |  |  |  |  | .561*** (.149) |  |  |  |  |
| ***Rangpur*** |  |  |  |  | -.275 (.162) |  |  |  |  |
| ***Sylhet*** |  |  |  |  | .424* (.166) |  |  |  |  |
| ***Khulna*** |  |  |  |  | .633*** (.168) |  |  |  |  |
| ***Chottogram*** |  |  |  |  | -1.52*** (.103) |  |  |  |  |
| ***Mymensingh*** |  |  |  |  | .545** (.173) |  |  |  |  |
| **Living place** |  |  |  |  |  |  |  |  |  |
| ***Rural (ref)*** |  |  |  |  |  |  |  |  |  |
| ***Urban*** |  |  |  |  |  | -.445*** (.094) |  |  |  |
| **Job** |  |  |  |  |  |  |  |  |  |
| ***Paid services (ref)*** |  |  |  |  |  |  |  |  |  |
| ***Business/Farming*** |  |  |  |  |  |  | .081 (.147) |  |  |
| ***Nonpaid services*** |  |  |  |  |  |  | .098 (.111) |  |  |
| ***Student*** |  |  |  |  |  |  | .364*** (.099) |  |  |
| **Frontliner** |  |  |  |  |  |  |  |  |  |
| ***No (ref)*** |  |  |  |  |  |  |  |  |  |
| ***Yes*** |  |  |  |  |  |  |  | .143 (.102) |  |
| **Marital status** |  |  |  |  |  |  |  |  |  |
| ***Unmarried (ref)*** |  |  |  |  |  |  |  |  |  |
| ***Married*** |  |  |  |  |  |  |  |  | -.494*** (.082) |
| **Constant** | 4.01*** (.057) | 5.12*** (.120) | 3.95*** (.068) | 4.09*** (.044) | 4.37*** (.057) | 4.44*** (.081) | 4.06*** (.071) | 4.19*** (.46) | 4.43*** (.054) |

Note. Dependent variable: Anxiety scores (square root transformed), **** *p*<0.0001, *** *p*<0.001, ** *p*<0.01, * *p*<0.05

**S2 Table. Univariable regression coefficients for modifiable factors associated with anxiety scores (square root transformed) among Bangladeshi adults**

| **Explanatory variables** | **Coefficient (Standard Error)** | **Coefficient (Standard Error)** | **Coefficient (Standard Error)** | **Coefficient (Standard Error)** | **Coefficient (Standard Error)** | **Coefficient (Standard Error)** | **Coefficient (Standard Error)** |
| --- | --- | --- | --- | --- | --- | --- | --- |
| **Socioeconomic status** | -.078** (.027) |  |  |  |  |  |  |
| **Covid-19 Infection (Self)** |  |  |  |  |  |  |  |
| ***No (ref)*** |  |  |  |  |  |  |  |
| ***Yes*** |  | .156 (.121) |  |  |  |  |  |
| **Covid-19 Infection (Family member)** |  |  |  |  |  |  |  |
| ***No (ref)*** |  |  |  |  |  |  |  |
| ***Yes*** |  |  | .211* (.082) |  |  |  |  |
| **Vaccination status (Self)** |  |  |  |  |  |  |  |
| ***No (ref)*** |  |  |  |  |  |  |  |
| ***Yes, one doze*** |  |  |  | -0.879*** (.224) |  |  |  |
| ***Yes, two dozes*** |  |  |  | -1.413*** (.109) |  |  |  |
| **Optimism** |  |  |  |  | -.192*** (.017) |  |  |
| **Pessimism** |  |  |  |  |  | .124*** (.018) |  |
| **Mindfulness** |  |  |  |  |  |  | -.109*** (.007) |
| **Constant** | 4.61*** (.142) | 4.20*** (.044) | 4.12*** (.055) | 4.61*** (.043) | 5.75*** (.143) | 3.45*** (.111) | 6.14*** (.124) |

Note. Dependent variable: Anxiety scores (square root transformed), *** *p*<0.001, ** *p*<0.01, * *p*<0.05

## Factors associated with depression scores

**S3 Table. Univariable regression coefficients for non-modifiable factors associated with depression scores (square root transformed) among Bangladeshi adults**

| **Explanatory variables** | **Coefficient (Standard Error)** | **Coefficient (Standard Error)** | **Coefficient (Standard Error)** | **Coefficient (Standard Error)** | **Coefficient (Standard Error)** | **Coefficient (Standard Error)** | **Coefficient (Standard Error)** | **Coefficient (Standard Error)** | **Coefficient (Standard Error)** |
| --- | --- | --- | --- | --- | --- | --- | --- | --- | --- |
| **Sex** |  |  |  |  |  |  |  |  |  |
| ***Male (ref)*** |  |  |  |  |  |  |  |  |  |
| ***Female*** | .088 (.062) |  |  |  |  |  |  |  |  |
| **Age (in years)** |  | -.033*** (.003) |  |  |  |  |  |  |  |
| **Education** |  |  |  |  |  |  |  |  |  |
| ***Postgraduate (ref)*** |  |  |  |  |  |  |  |  |  |
| ***Nonformal*** |  |  | .007 (.156) |  |  |  |  |  |  |
| ***SSC/Equivalent*** |  |  | .180 (.128) |  |  |  |  |  |  |
| ***HSC/Equivalent*** |  |  | .672*** (.077) |  |  |  |  |  |  |
| ***Bachelor/Equivalent*** |  |  | .472*** (.078) |  |  |  |  |  |  |
| **History of mental illness** |  |  |  |  |  |  |  |  |  |
| ***No (ref)*** |  |  |  |  |  |  |  |  |  |
| ***Yes*** |  |  |  | .695*** (.095) |  |  |  |  |  |
| ***Prefer Not to Say*** |  |  |  | .606*** (.168) |  |  |  |  |  |
| **Division** |  |  |  |  |  |  |  |  |  |
| ***Dhaka (ref)*** |  |  |  |  |  |  |  |  |  |
| ***Barisal*** |  |  |  |  | .070 (.122) |  |  |  |  |
| ***Rajshahi*** |  |  |  |  | .494*** (.115) |  |  |  |  |
| ***Rangpur*** |  |  |  |  | -.220 (.125) |  |  |  |  |
| ***Sylhet*** |  |  |  |  | .020 (.128) |  |  |  |  |
| ***Khulna*** |  |  |  |  | .081 (.129) |  |  |  |  |
| ***Chottogram*** |  |  |  |  | -1.096*** (.080) |  |  |  |  |
| ***Mymensingh*** |  |  |  |  | .154 (.134) |  |  |  |  |
| **Living place-urban** |  |  |  |  |  |  |  |  |  |
| ***Rural (ref)*** |  |  |  |  |  |  |  |  |  |
| ***Urban*** |  |  |  |  |  | -.227** (.071) |  |  |  |
| **Job** |  |  |  |  |  |  |  |  |  |
| ***Paid services (ref)*** |  |  |  |  |  |  |  |  |  |
| ***Business/Farming*** |  |  |  |  |  |  | .273* (.109) |  |  |
| ***Nonpaid services*** |  |  |  |  |  |  | .308*** (.111) |  |  |
| ***Student*** |  |  |  |  |  |  | .652*** (.074) |  |  |
| **Frontliner** |  |  |  |  |  |  |  |  |  |
| ***No (ref)*** |  |  |  |  |  |  |  |  |  |
| ***Yes*** |  |  |  |  |  |  |  | .015 (.077) |  |
| **Marital status** |  |  |  |  |  |  |  |  |  |
| ***Unmarried (ref)*** |  |  |  |  |  |  |  |  |  |
| ***Married*** |  |  |  |  |  |  |  |  | -.582*** (.061) |
| **Constant** | 2.69*** (.043) | 3.75*** (.089) | 2.41*** (.051) | 2.63*** (.033) | 2.90*** (.044) | 2.91*** (.062) | 2.42*** (.053) | 2.73*** (.035) | 2.99*** (.040) |

Note. Dependent variable: depression scores (square root transformed), **** *p*<0.0001, *** *p*<0.001, ** *p*<0.01, * *p*<0.05

**S4 Table. Univariable regression coefficients for modifiable factors associated with depression scores (square root transformed) among Bangladeshi adults**

| **Explanatory variables** | **Coefficient (Standard Error)** | **Coefficient (Standard Error)** | **Coefficient (Standard Error)** | **Coefficient (Standard Error)** | **Coefficient (Standard Error)** | **Coefficient (Standard Error)** | **Coefficient (Standard Error)** |
| --- | --- | --- | --- | --- | --- | --- | --- |
| **Socioeconomic status** | -.096*** (.020) |  |  |  |  |  |  |
| **Covid-19 Infection (Self)** |  |  |  |  |  |  |  |
| ***No (ref)*** |  |  |  |  |  |  |  |
| ***Yes*** |  | .034 (.091) |  |  |  |  |  |
| **Covid-19 Infection (Family member)** |  |  |  |  |  |  |  |
| ***No (ref)*** |  |  |  |  |  |  |  |
| ***Yes*** |  |  | .211** (.062) |  |  |  |  |
| **Vaccination status (Self)** |  |  |  |  |  |  |  |
| ***No (ref)*** |  |  |  |  |  |  |  |
| ***Yes, one doze*** |  |  |  | -0.684*** (.169) |  |  |  |
| ***Yes, two dozes*** |  |  |  | -1.135*** (.082) |  |  |  |
| **Optimism** |  |  |  |  | -.167*** (.013) |  |  |
| **Pessimism** |  |  |  |  |  | .071*** (.014) |  |
| **Mindfulness** |  |  |  |  |  |  | -.105*** (.004) |
| **Constant** | 3.23*** (.107) | 2.73*** (.033) | 4.12*** (.055) | 2.93*** (.033) | 4.07*** (.107) | 2.32*** (.084) | 4.59*** (.089) |

Note. Dependent variable: depression scores (square root transformed), *** *p*<0.001, ** *p*<0.01, * *p*<0.05

## Factors associated with stress scores

**S5 Table. Univariable regression coefficients for non-modifiable factors associated with stress scores among Bangladeshi adults**

| **Explanatory variables** | **Coefficient (Standard Error)** | **Coefficient (Standard Error)** | **Coefficient (Standard Error)** | **Coefficient (Standard Error)** | **Coefficient (Standard Error)** | **Coefficient (Standard Error)** | **Coefficient (Standard Error)** | **Coefficient (Standard Error)** | **Coefficient (Standard Error)** |
| --- | --- | --- | --- | --- | --- | --- | --- | --- | --- |
| **Sex** |  |  |  |  |  |  |  |  |  |
| ***Male (ref)*** |  |  |  |  |  |  |  |  |  |
| ***Female*** | .480** (.146) |  |  |  |  |  |  |  |  |
| **Age (in years)** |  | -.024*** (.007) |  |  |  |  |  |  |  |
| **Education** |  |  |  |  |  |  |  |  |  |
| ***Postgraduate (ref)*** |  |  |  |  |  |  |  |  |  |
| ***Nonformal*** |  |  | .851* (.373) |  |  |  |  |  |  |
| ***SSC/Equivalent*** |  |  | .484 (.305) |  |  |  |  |  |  |
| ***HSC/Equivalent*** |  |  | 1.082*** (.183) |  |  |  |  |  |  |
| ***Bachelor/Equivalent*** |  |  | .663*** (.187) |  |  |  |  |  |  |
| **History of mental illness** |  |  |  |  |  |  |  |  |  |
| ***No (ref)*** |  |  |  |  |  |  |  |  |  |
| ***Yes*** |  |  |  | .983*** (.227) |  |  |  |  |  |
| ***Prefer Not to Say*** |  |  |  | 1.263** (.400) |  |  |  |  |  |
| **Division** |  |  |  |  |  |  |  |  |  |
| ***Dhaka (ref)*** |  |  |  |  |  |  |  |  |  |
| ***Barisal*** |  |  |  |  | -.054 (.307) |  |  |  |  |
| ***Rajshahi*** |  |  |  |  | .444 (.289) |  |  |  |  |
| ***Rangpur*** |  |  |  |  | -.275 (.315) |  |  |  |  |
| ***Sylhet*** |  |  |  |  | .135 (.321) |  |  |  |  |
| ***Khulna*** |  |  |  |  | .591 (.325) |  |  |  |  |
| ***Chottogram*** |  |  |  |  | -.045 (.201) |  |  |  |  |
| ***Mymensingh*** |  |  |  |  | -.114 (.337) |  |  |  |  |
| **Living place** |  |  |  |  |  |  |  |  |  |
| ***Rural (ref)*** |  |  |  |  |  |  |  |  |  |
| ***Urban*** |  |  |  |  |  | -.115 (.169) |  |  |  |
| **Job** |  |  |  |  |  |  |  |  |  |
| ***Paid services (ref)*** |  |  |  |  |  |  |  |  |  |
| ***Business/Farming*** |  |  |  |  |  |  | .635* (.260) |  |  |
| ***Nonpaid services*** |  |  |  |  |  |  | 1.223*** (.196) |  |  |
| ***Student*** |  |  |  |  |  |  | 1.184*** (.176) |  |  |
| **Frontliner** |  |  |  |  |  |  |  |  |  |
| ***No (ref)*** |  |  |  |  |  |  |  |  |  |
| ***Yes*** |  |  |  |  |  |  |  | -.770*** (.182) |  |
| **Marital status** |  |  |  |  |  |  |  |  |  |
| ***Unmarried (ref)*** |  |  |  |  |  |  |  |  |  |
| ***Married*** |  |  |  |  |  |  |  |  | -.711*** (.147) |
| **Constant** | 7.77*** (.102) | 8.72*** (.217) | 7.46*** (.051) | 7.85*** (.079) | 7.96*** (.110) | 7.92*** (.146) | 7.26*** (.125) | 8.16*** (.081) | 8.31*** (.096) |

Note. Dependent variable: stress scores, **** *p*<0.0001, *** *p*<0.001, ** *p*<0.01, * *p*<0.05

**S6 Table. Univariable regression coefficients for modifiable factors associated with stress scores among Bangladeshi adults**

| **Explanatory variables** | **Coefficient (Standard Error)** | **Coefficient (Standard Error)** | **Coefficient (Standard Error)** | **Coefficient (Standard Error)** | **Coefficient (Standard Error)** | **Coefficient (Standard Error)** | **Coefficient (Standard Error)** |
| --- | --- | --- | --- | --- | --- | --- | --- |
| **Socioeconomic status** | -.311*** (.047) |  |  |  |  |  |  |
| **Covid-19 Infection (Self)** |  |  |  |  |  |  |  |
| ***No (ref)*** |  |  |  |  |  |  |  |
| ***Yes*** |  | .023 (.216) |  |  |  |  |  |
| **Covid-19 Infection (Family member)** |  |  |  |  |  |  |  |
| ***No (ref)*** |  |  |  |  |  |  |  |
| ***Yes*** |  |  | .277^†^ (.147) |  |  |  |  |
| **Vaccination status (Self)** |  |  |  |  |  |  |  |
| ***No (ref)*** |  |  |  |  |  |  |  |
| ***Yes, one doze*** |  |  |  | -0.264 (.417) |  |  |  |
| ***Yes, two dozes*** |  |  |  | -.776*** (.203) |  |  |  |
| **Optimism** |  |  |  |  | -.484*** (.030) |  |  |
| **Pessimism** |  |  |  |  |  | -.111** (.032) |  |
| **Mindfulness** |  |  |  |  |  |  | -.243*** (.011) |
| **Constant** | 9.56*** (.251) | 8.00*** (.078) | 7.88*** (.098) | 8.13*** (.081) | 11.85*** (.247) | 8.65*** (.200) | 12.27*** (.212) |

Note. Dependent variable: stress scores, *** *p*<0.001, ** *p*<0.01, * *p*<0.05, † *p*=.06
