# Supplementary material for: Modifiable and nonmodifiable factors associated with anxiety, depression, and stress after one year of the COVID-19 pandemic
Source: PLoS One. 2023 Mar 23;18(3):e0283422. doi: 10.1371/journal.pone.0283422 (PMC10035880; doi:10.1371/journal.pone.0283422)
Supplement: Attachment — Submitted filename: Response to reviewers (PONE-D-22-07194).docx [file pone.0283422.s002.docx]

Response to reviewers

PONE-D-22-07194

Modifiable and nonmodifiable factors associated with anxiety, depression, and stress after one year of the COVID-19 pandemic

| **SN** | **Reviewers’ comment** | **Authors’ response** | **In-text edition** |
| --- | --- | --- | --- |
| 1 | The authors provide an interesting report on a study interested in modifiable and non-modifiable factors associated with stress, anxiety and depression during the pandemic. The study was conducted in Bangladesh in May and June 2021, at a time most restriction were lifted, although new lockdowns occurred in some regions starting June 22. | Thank you for summarizing our work. |  |
| 2 | Introduction: The introduction makes a good job at examining the prevalence of mental health issues during the pandemic. But it could be expected that the presented literature includes references related to depression and anxiety during past similar challenges (epidemic/pandemic). Framed as it is, the study doesn't present an ambition towards generalization to future events. | Literature on psychological reactions caused by the past pandemics has been included. | Page 4, line 65-74 |
| 3 | Lines 65 - 74: The authors report on 3 studies that were conducted in Bangladesh in relation to stress depression and anxiety during the pandemic in 2020. Were other studies conducted since? | There were a few other studies with small samples but those studied used various assessment methods, hence, they were incomparable to our data. | NA |
| 4 | The authors should clearly define what they consider modifiable and non-modifiable factors associated with mental health (they start doing so line 125) and frame their literature review from this perspective as this is an important part of their study. From reading the manuscript from beginning to end, I understand that mindfulness, optimism and pessimism are considered modifiable factors, whereas all other variables are not. This should be made clearer from the beginning. | The definitions of modifiable and non-modifiable factors have been clarified. | Line 94-98 |
| 5 | It would be helpful for the readers that the authors included hypotheses at the end of the introduction, rather than the general aim to investigate "modifiable and non-modifiable factors" associated with mental health. | Three hypotheses have been added at the end of the Introduction. | Line 149-157 |
| 6 | Methods: Lines 203: The authors report the use of an unreliable instrument. The pessimism scale, with an alpha of .36 should be removed from the analyses. The optimism scale, with an alpha of .59, has a reliability close to acceptable. I suggest it remains used unless another review considers this a more important flaw. | Pessimism scale has been removed | Line 230-240 |
| 7 | Lines 245 to 247. The authors use a median score to determine whether participants suffered from stress. From this operationalization, the prevalence of stress is by definition close to 50%. | Due to lack of established cut-off point for the stress (PSS-4) scale, the classification has been removed from the analysis. | NA |
| 8 | The authors should indicate which statistical analyses were performed for each variable and justify their choice. | Clarification and justification for the choice of tests | Line 281-314 |
| 9 | Lines 248 ++, they mention the use of regressions, yet t tests are presented at the beginning of the results section. If this is possible, a useful variable to include would be whether participants were living in a region that was in lockdown at the time of their participation. | The choice of tests has been clarified.  During the time of data collection, all Bangladesh divisions were under strict lockdown. | NA |
| 10 | Results: Line 279. The variable "mental health status" has not been described before its use. | This section has been revised in sync with the objectives. | NA |
| 11 | Lines 280 ++: The authors report on different statistical tests that should be presented in light of the authors research questions or relate to the sample description. The authors could start by mentioning something in the lines of "To examine modifiable and nonmodifiable factors associated with the considered mental health outcomes, we tested the association of XXXX with YYYY. | Clarification and justification for the choice of tests | Line 281-214 |
| 12 | For the tests related to the description of the sample, this should be better presented in the methods section - preferably in a table, examples of these are history of mental disorder, occupation, vaccination status, recovery from COVID etc. In any case the description of the sample (with or without tests) should be reported in the methods section. | A separate table (Table 1) has been created to describe sample characteristics. This table has been placed in the Method section. | Line 206 |
| 13 | For instance see Table 1 in Varma, P., Junge, M., Meaklim, H., & Jackson, M. L. (2021). Younger people are more vulnerable to stress, anxiety and depression during COVID-19 pandemic: A global cross-sectional survey. Progress in Neuro-Psychopharmacology and Biological Psychiatry, 109, 110236. <https://www.sciencedirect.com/science/article/pii/S0278584620305522>? | Thank you for the recommendation |  |
| 14 | Table 1 (in the authors manuscript): What are the tests performed (please present these in the methods section, with justification)? The coefficients / test statistics should be presented in the table, not only the p-values. | Test statistic has been added in Table 2 (previously Table 1) | Line 357-358 |
| 15 | Line 345++: Again, the prevalence of high stress directly derives from the authors' use of a median split. This is not informative. | Due to lack of established cut-off point for the stress (PSS-4) scale, the classification has been removed from the analysis. | NA |
| 16 | The structure of the results section could be made more familiar by using a more frequently used organization in clinical psychology. | The structure of the result section has been reorganized and clarified. | NA |
| 17 | Discussion: The authors could start by reminding the readers of their research questions. My opinion is the authors could try to relate their study to similarly stressful situation as the current pandemic (past research + try to provide implication for future situations), thereby making it more relevant for researchers interested in future stressors. | The discussion has been revised thoroughly. | NA |
| 18 | The authors should revise the language to improve readability. Only some examples are indicated below. I suggest the authors use proofreading services. Lines 75, 76: Repharse "panicking too many people" (makes it seem there is an appropriate amount) Lines 81 - 82: Rephrase "not equally lethal to all; some still maintained a healthy state" (lethal means deadly) Lines 154-155: Rephrase "19 vital questions in the questionnaire" (vital means required to live) Through out the manuscript: please use "mental disorder" instead of "mental illness" Line 433: Rephrase 'mimic' (suggests the prevalence rates are capable of perceiving rates in earlier studies and to adapt to those) | The manuscript has been thoroughly proofread by a colleague. | NA |
