# Supplementary material for: Modifiable and nonmodifiable factors associated with anxiety, depression, and stress after one year of the COVID-19 pandemic
Source: PLoS One. 2023 Mar 23;18(3):e0283422. doi: 10.1371/journal.pone.0283422 (PMC10035880; doi:10.1371/journal.pone.0283422)
Supplement: Attachment — Submitted filename: Response to reviewers R 2 (PONE-D-22-07194).docx [file pone.0283422.s003.docx]

Response to reviewers (R 2)

PONE-D-22-07194

Modifiable and nonmodifiable factors associated with anxiety, depression, and stress after one year of the COVID-19 pandemic

| **SN** | **Reviewers’ comment** | **Author’s response** | **In-text edition** |
| --- | --- | --- | --- |
|  | Reviewer 2 |  |  |
| 1 | Thank you for invitation to review Modifiable and nonmodifiable factors associated with anxiety, depression, and stress after one year of the COVID-19 pandemic. The manuscript was clear, and methodologies intact and add value to the literature. | We appreciate the reviewer’s feedback. | NA |
| 2 | Here are minor changes requested to improve.  Introduction:  To long (1190), need to reduce it to be with the 700 words only.  Method  Sample description:  This section should be at result section | The placement of the sample description does not have a fixed position, with authors using both the method and result section. Reviewer(s) in the first round suggested placing it in the method section, which we find justifiable as the result section of this paper is lengthy. | NA |
| 3 | General Comments:  All other section was clear and straight forward. | Thank you very much. | NA |
|  | Review 2 |  |  |
| 5 | The authors of the article conducted a study to investigate the predictors of anxiety, depression, and stress among Bangladeshi adults one year into the COVID-19 pandemic. The study recruited 1897 participants and used various self-reported measures to assess mental health outcomes, mindfulness, and optimism. My appreciation for successful completion of the study. | We thank you for your appreciation. | NA |
|  | The changes in the articles after the first reviewer’s comments, as shown in the Revision 1 manuscript, solve a majority of the issues in the article. | Indeed, the first round of review was thorough. | NA |
|  | My comment would be, to include more recent studies in the discussion section. A tabulated or textual representation and comparison of all recent studies exploring the mental health implications and its predictors would add more meaning to the discussion section. Articles after 2021 have not been highlighted. I understand that this article restricts itself to 1-year post-pandemic but, it would be informative to explore the post covid angle in more recent publications as well.  Also, a comparison of the situation of High-income countries and Low and middle-income countries would be interesting. At least a few sentences with meaningful references would be good. | Thank you for your suggestion. We have now revised the entire discussion with the latest evidence. Also, the discussion covers a comparison of countries with diverse incomes. |  |
|  |  |  | Page 444-472 |
